# Prenatal Diagnosis of Aberrant Right Subclavian Artery: Association with Genetic Abnormalities

**DOI:** 10.1055/s-0041-1732461

**Published:** 2021-07-27

**Authors:** Cátia Sofia Ferreira Pinto Lourenço, Ana Luísa Carriço, Francisco Manuel da Silva Valente

**Affiliations:** 1 Centro Hospitalar de Vila Nova de Gaia/Espinho, Vila Nova de Gaia, Portugal

**Keywords:** aberrant right subclavian artery, prenatal diagnosis, screening, genetic abnormalities, artéria subclávia direita aberrante, diagnóstico pré-natal, triagem, anormalidades genéticas

## Abstract

**Objective** The objective of the present study was to determine the frequency of malformations and chromosomal abnormalities in a population of fetuses with an aberrant right subclavian artery (ARSA).

**Methods** This is a 6-year retrospective study of fetuses with a prenatal diagnosis of ARSA conducted during the period between September 2013 and June 2019 at a fetal medicine unit. Data were collected from ultrasound, fetal echocardiograms, genetic studies, and neonatal records.

**Results** An ARSA was diagnosed in 22 fetuses. An ARSA was an isolated finding in 18 out of 22 cases (82%). Associated abnormal sonographic findings were found in 4 cases. All cases underwent invasive testing. In 1 of the cases, a chromosomal abnormality was detected (mos 45,X [13]/46,X,e(X) (p22.1q22.1)). No cases of congenital heart disease were found in any of these fetuses. There were two cases in which the postnatal evaluation revealed a malformation: one case of hypospadias and 1 case of cleft palate.

**Conclusion** The presence of an isolated ARSA is benign and is not associated with chromosomal abnormalities. The finding of ARSA, however, warrants a detailed fetal ultrasound in order to exclude major fetal abnormalities and other soft markers.

## Resumo

**Objetivo** O objetivo do presente estudo foi determinar a frequência de malformaçães e anomalias cromossômicas em uma população de fetos com artéria subclávia direita aberrante (ARSA).

**Métodos** Este é um estudo retrospectivo de 6 anos de fetos com diagnóstico pré-natal de ARSA realizado durante o período de setembro de 2013 a junho de 2019 em uma unidade de medicina fetal. Os dados foram coletados de ultrassom, ecocardiograma fetal, estudos genéticos e registros neonatais.

**Resultados** Um ARSA foi diagnosticado em 22 fetos. Um ARSA foi um achado isolado em 18 dos 22 casos (82%). Achados ultrassonográficos anormais associados foram encontrados em 4 casos. Todos os casos foram submetidos a testes invasivos. Em um dos casos, foi detectada uma anormalidade cromossômica (mos 45, X [13] / 46, X, e (X) (p22.1q22.1)). Nenhum caso de doença cardíaca congênita foi encontrado em qualquer um desses fetos. Houve dois casos em que a avaliação pós-natal revelou a malformação: um caso de hipospádia e 1 caso de fenda palatina.

**Conclusão** A presença de ARSA isolado é benigna e não está associada a anormalidades cromossômicas. O achado de ARSA, no entanto, justifica uma ultrassonografia fetal detalhada para excluir anormalidades fetais importantes e outros marcadores leves.

## Introduction

An abnormal origin of the right subclavian artery (RSA) is the most common aortic branching abnormality, and it has been reported postnatally in ∼ 1 to 2% in the general population in autopsy series. 1 2 3 4 5 6 In contrast to the normal aortic arch branching pattern, in which the right subclavian artery branches off the brachiocephalic trunk, an aberrant right subclavian artery (ARSA) arises as a 4 ^th^ aortic arch vessel and passes behind the trachea and the esophagus and courses to the right arm. 7 8 9 10 11

Being usually asymptomatic and considered as a normal variant, an ARSA can sometimes cause clinical symptoms due to its trajectory behind the trachea and the esophagus (dysphagia, cough, and dyspnea). 8 9 10 11 In the postnatal period, and, most recently, also in the prenatal period, ARSA was found significantly more often in subjects with congenital heart disease 12 or with chromosomal abnormalities, particularly trisomy 21, 2 13 with the relative risk multiplied by 3.94. 14 However, most fetuses with trisomy 21 have additional anatomic features in addition to the ARSA. 3 5 8 13 An ARSA has also been reported in fetuses with other, less common, genetic anomalies. 5 6 15 16 17 18 19 20 21 Some authors recommend invasive testing even if an ARSA is isolated. 7 However, more recent studies did not find an association between isolated ARSA and chromosomal abnormalities and, therefore, do not recommend invasive testing in these cases. 19 20 21 22

The aim of our study was to determine the frequency and the nature of associated anomalies, such as malformations and chromosomal abnormalities, in a population of fetuses diagnosed with an ARSA through screening or diagnostic ultrasound, and to assess the postnatal outcome.

## Methods

An ARSA was prospectively sought in all patients who underwent obstetric ultrasound during the 2 ^nd^ trimester of gestation. The examinations were performed using Voluson E8 Expert ultrasound devices (GE Healthcare, Chicago, IL, USA) by a transabdominal approach between August 2013 and June 2019 by 6 sonographers experts in obstetrics ultrasonography. They included all patients referred for 2 ^nd^ trimester ultrasound in our department, including high- and low-risk pregnancies. During fetal heart assessment, the course of the RSA was observed after the assessment of 4-chamber view, outflow tracts and the 3 vessel and trachea view according to the technique described by Chaoui et al. 2 In addition to the B-mode segmental view approach, color Doppler ultrasonography was used for visualizing the transverse 3-vessel and tracheal view. The normal RSA in the axial plane was visualized as an S-shaped vessel passing anterior to the trachea at the clavicle level. An ARSA was detected as a vessel arising separately from the junction of the aortic arch and ductus arteriosus and having a retrotracheal course toward the right arm. The course of the ARSA was straight, without an S-shape proximal concavity surrounding the trachea anteriorly. All cases were referred for fetal echocardiogram performed by a pediatric cardiologist. The cases of ARSA were categorized as isolated if ARSA was the only sonographic finding, and as nonisolated in cases of associated ultrasound abnormalities or 2 ^nd^ trimester soft markers. Soft markers included the sonographic findings associated with an increased risk of chromosomal abnormality – increased nuchal fold, nasal bone hypoplasia, echogenic bowel, echogenic intracardiac focus, choroid plexus cyst, pyelectasis and femur or humerus length < 5 ^th^ centile. After the diagnosis, patients were offered invasive testing by amniocentesis. Until 2018, karyotype and fluorescence in situ hybridization (FISH) for 22q11.2 microdeletion were offered, and after 2018, QF-PCR and cGH-array were offered. Follow-up scans were performed in all cases in which the pregnancy continued. An ARSA was not evaluated postnatally by imaging in liveborn infants, while it was systematically investigated in cases of termination of pregnancy. Outcomes were collected from all ARSA fetuses from hospital records.

## Results

**Fig. 1:**
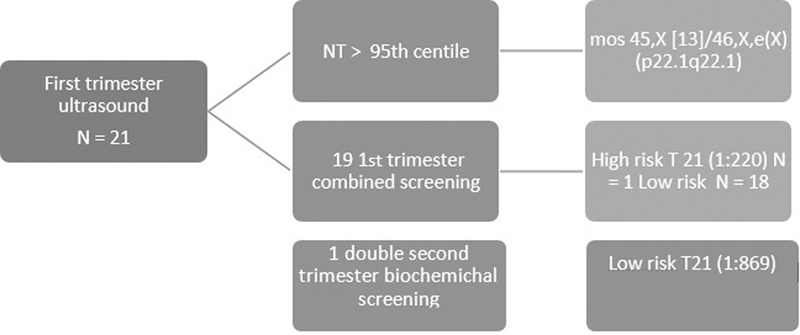
Results of first trimester screening in fetus with ARSA. NT = nuchal translucency; 1 ^st^ = first; T21 = trisomy 21.

Between August 2013 and June 2019, an ARSA was diagnosed in 22 fetuses in the 2 ^nd^ trimester (between 20 and 22 weeks). A total of 8,699 second trimester ultrasounds were done, resulting in a prevalence of 0.25%. The mean maternal age was 29 years old (range 18–38 years old). The fetal gender was mainly female. Only 3 out of 22 ARSA cases were male. An ARSA was an isolated finding in 18 out of 22 cases (82%). An ARSA was associated with other sonographic findings in the remaining 4 out of 22 cases (18%). A total of 21 cases underwent 1 ^st^ trimester screening ultrasound, 19 cases underwent 1 ^st^ trimester combined screening, and 1 case underwent 2 ^nd^ trimester screening. Of these, there were 18 cases of 1 ^st^ trimester low-risk screening and 1 case of increased risk for trisomy 21 (1:220). There was 1 case with nuchal translucency > 95 ^th^ centile, and the patient opted for invasive testing (chorionic villous sampling) ( Fig. 1 ).

There were no cases of associated congenital heart disease. There were 3 cases of associated ultrasound signs in the 2 ^nd^ trimester: 1 case of choroid plexus cysts, 1 case of a megacisterna magna, and 1 case with measurements of long bones < 5 ^th^ centile and head circumference < 5 ^th^ centile. There was 1 case of head circumference < 5 ^th^ centile in the 3 ^rd^ trimester. The ARSA was apparently isolated in 18 cases.

Fetal genetic testing was systematically proposed and analyzed in all fetuses (karyotype and FISH for 22q11.2 microdeletion or QF-PCR and cGH array) during the prenatal period.

Genetic testing proved to be normal for 21 out of 22 (95%) and abnormal for 1 out of 22 fetuses (5%). This case had nuchal translucency > 95 ^th^ centile in the 1 ^st^ trimester and measurements of long bones < 5 ^th^ centile and head circumference < 5 ^th^ centile in the second trimester, having an unusual abnormality, such as mos 45,X [13]/46,X,e(X) (p22.1q22.1). No chromosomal abnormalities were detected in fetuses with an isolated ARSA. There were no cases in this population of trisomy 21 or 22q11.2 microdeletion.

In what concerns obstetrics outcomes, there was 1 case of termination of pregnancy, 1 case of intrauterine fetal demise at 32 weeks, and 2 cases of head circumference below the 5 ^th^ centile in the 3 ^rd^ trimester whose fetal cerebral magnetic resonance imaging (MRI) were normal.

On ongoing pregnancies, there were two cases of late preterm birth. All the remaining cases reached full term. There were 2 cases of birthweight < 2500 g, corresponding to the 2 preterm births.

All neonates were examined postnatally, and two congenital anomalies with no prenatal diagnosis were seen: one case of hypospadias and one case of cleft palate. All born children had normal development ( Table 1 )

**Table 1:** Outcomes in fetuses with ARSA

Case	First trimester screening	Method of diagnosis	Fetal Karyotype	Associated abnormalities/soft markers	Outcome
1	NT > 95 ^th^ centile	CVS and amniocentesis	mos 45,X [13]/46,X,e(X) (p22.1q22.1)	long bones < 5 ^th^ centile and head circumference < 5 ^th^ centile	TOP at 23 weeks
2	Low risk	amniocentesis	Normal	Choroid plexus cyst	Balanic Hypospadias Normal development
3	Low risk	amniocentesis	Normal	Megacysterna magna	Normal fetal cerebral MRI Normal development
4	High risk (1:220 T21)	Amniocentesis	Normal	None	Normal development
5	Low risk	Amniocentesis	Normal	Head circumference < 5 ^th^ centile in the 3 ^rd^ trimester	Normal fetal cerebral MRI Normal development
6	Low risk	Amniocentesis	Normal	none	Intrauterine fetal demise at 32 weeks Autopsy: ARSA, subendocardic elastosis; fetal distress
7	Low risk	Amniocentesis	Normal	None	Postnatal diagnosis of cleft palate. Normal development

Abbreviations: CVS, chorionic villous sampling; MRI, magnetic resonance imaging; NT, nuchal translucency; T21, trisomy 21; TOP, termination of pregnancy.

## Discussion

The presence of an ARSA in fetuses with Down syndrome was described for the first time by Chaoui et al. 2 In their preliminary study, they identified an ARSA in 35.7% of fetuses with Down syndrome in the 2 ^nd^ and 3 ^rd^ trimester. Since then, several studies reported that an ARSA was one of the most powerful independent markers for Down syndrome.

Chaoui et al. 2 found a fetus with trisomy 21 in whom the only ultrasonographic abnormality was an ARSA. In this case, the maternal age was 42 years old, the nuchal translucency thickness was < 95th percentile, and the result of serum markers was not mentioned. It is very likely that fetal karyotyping was performed because of the initially high risk of aneuploidy.

In the study by Borenstein et al., 5 an ARSA was also isolated in one fetus with Down syndrome. However, the population included in their study was at a high risk of chromosomal abnormality.

Rembouskos et al. 6 revealed 2 chromosomal abnormalities in fetuses with isolated ARSAs: in a fetus with trisomy 21, the 1 ^st^ trimester combined risk was 1/39, and in a fetus with a trisomy 21 mosaicism, the combined risk was 1/402. Cardiovascular defects were the most frequently associated abnormality in euploid fetuses. Therefore, the authors concluded that fetal echocardiography should be offered in all cases of ARSA.

Paladini et al. 13 found 8 fetuses carrying trisomy 21 with an isolated ARSA, and ARSA revealed as the one of the most important 2 ^nd^ trimester marker for Down syndrome together with hypoplasic nasal bone and increased nuchal fold. However, at that time, the standard combined 1 ^st^ -trimester screening test was not applied routinely, so there is no information regarding 1 ^st^ trimester risk in the apparently isolated cases of ARSA.

In the study by Esmer et al., 7 6 fetuses with trisomy 21 were classified as having isolated ARSA. A review of these cases showed that in 4 out 6 of these patients the combined 1 ^st^ trimester screening was high risk for trisomy 21, and in 2 out of 6 patients the 1 ^st^ trimester risk was not evaluated; these 2 patients were 37 and 38 years old.

Gursoy Erzincan et al. 9 found a weak association with Down syndrome in a low-risk population. An ARSA is more commonly detected in fetuses with Down syndrome than in euploid fetuses, and, in most cases, it is associated with other pathologic sonographic findings. The authors conclude that ARSA by itself does not create a sufficient indication for invasive testing.

Fehmi Yazicioğlu et al. 19 studied the prevalence of an ARSA in a mixed population and found a prevalence rate of 1.1%. However, the study was composed of high-risk pregnancies, owing to the high incidence of Down syndrome in their study population.

In a meta-analysis, Agathokleous et al. 14 reported that an ARSA increased the Down syndrome risk by 3.94, and emphasized that most of the studies included in the meta-analysis were performed in high-risk pregnancies.

Considering the meta-analysis by De León-Luis et al., 16 we must differentiate isolated from non-isolated ARSAs. The ARSAs detected among cases of Down syndrome were all associated with other markers of trisomy 21. They did not find any correlation between an isolated ARSA and Down syndrome.

Scala et al., 4 in a systematic review and meta-analysis evaluating an ARSA in fetuses with Down syndrome, showed that an ARSA is a clinically important marker of trisomy 21, but not sufficient to recommend fetal karyotyping in isolated cases.

In recent studies, no cases of Down syndrome or pathogenic copy number variants were reported in fetuses with an isolated ARSA 4 16 19 20 21 22 Svirsky et al. 20 report the findings of chromosomal microarray analysis in 62 fetuses referred for genetic counseling due to the finding of ARSA. In the 41 patients with isolated ARSA, no cases of trisomy 21 or any other chromosomal aberration were found. Maya et al. 21 showed that in 36 fetuses with isolated ARSA, pathogenic copy number variants were not found. These studies reported that all Down syndrome cases with an ARSA were associated with other markers. Sagi-Dain et al. 22 report the results of chromosomal microarray analysis in 246 fetuses with isolated ARSA. In 1 case, a trisomy 21 was detected, but this frequency did not significantly differ from the control population. In this report, there is no reference to the results of 1 ^st^ trimester screening in these cases. Aberrant right subclavian artery has a female predominance, 6 11 and our results also reflected a higher incidence of ARSA in females than in males. Some studies do not support this female predominance.

The present study has some limitations. Aberrant right subclavian artery was routinely searched only in 2 ^nd^ trimester ultrasound. This might have resulted in a lower incidence than that reported by previous studies.

Moreover, there was no systematic postnatal verification of the presence of an ARSA. The course of the RSA is not readily detectable by postnatal echocardiography, while other postnatal tests, such as MRI, are nonjustifiable in the absence of specific indications. Therefore, the only way of confirming the course of the RSA in asymptomatic cases remains fetal echocardiography. As is well known, the accuracy of fetal echocardiography at 20 to 22 weeks of gestation is well documented, so that we took the midtrimester scan as the gold standard. 6 In our series, fetal echocardiography has confirmed the initial diagnosis of ARSA, which underlines that, once the specific landmarks described are observed, a straightforward diagnosis of ARSA can be achieved. 6

## Conclusion

The conflicting evidence in the literature regarding the association of ARSA and chromosomal abnormalities is probably because the earlier studies did not differentiate between isolated ARSA and ARSA with additional ultrasound findings. The analysis of the literature combined with the results of our study suggest that in patients in whom the combined risk of chromosomal abnormalities in the 1 ^st^ trimester was evaluated, the presence of an isolated ARSA is a condition rarely associated with a chromosomal abnormality. In the case of an isolated ARSA, an ultrasound scan must be performed in a reference center, and especially an echocardiography, to confirm that there is no associated anomaly.
